# Transcriptome Profiling of *Ornithogalum dubium* Leaves and Flowers to Identify Key Carotenoid Genes for CRISPR Gene Editing

**DOI:** 10.3390/plants9040540

**Published:** 2020-04-21

**Authors:** Zunzheng Wei, Tzahi Arazi, Nofar Hod, Matat Zohar, Tal Isaacson, Adi Doron-Faigenboim, Noam Reznik, Iris Yedidia

**Affiliations:** 1 Institute of Plant Science, The Volcani Center, Agricultural Research Organization, Bet Dagan 50250, Israel; weizunzheng@163.com (Z.W.); tarazi@volcani.agri.gov.il (T.A.); tinka-4@hotmail.com (N.H.); adif@volcani.agri.gov.il (A.D.-F.); noamr@volcani.agri.gov.il (N.R.); 2 Newe Yaar Research Center, Agricultural Research Organization, Ramat Yishay 30095, Israel; matat@volcani.agri.gov.il (M.Z.); tali@volcani.agri.gov.il (T.I.)

**Keywords:** carotenoid pathway, ornamental, *Ornithogalum dubium*, transcriptome

## Abstract

*Ornithogalum dubium* is a popular ornamental monocot native to South Africa with flower colors ranging from pure white to deep orange. Gene editing based on the CRISPR/Cas9 system has recently been shown to hold potential for color improvement in ornamental flower crops. To apply this approach to *Ornithogalum* color manipulation, genomic or transcriptomic data must first be collected. Here, cDNA libraries of *O. dubium* leaves and flowers were constructed and sequenced using the Illumina HiSeq 2500. Over 155 million 100-bp paired-end reads were assembled into a transcriptome database of 360,689 contigs, of which 18,660 contigs were differentially expressed between leaves and flowers. Carotenoids are the main pigment imparting spectrum of orange hues to *O. dubium* flowers. By querying our database, we identified a total of 16 unique transcripts (unigenes) predicted to be involved in the carotenoid biosynthesis pathway of *Ornithogalum*. Combining carotenoid profiles, we further inferred several key unigenes responsible for floral coloration and accumulation in *O. dubium*, of which the gene LCYB/comp146645_c0 was found as a suitable target to generate potentially red flower varieties of *O. dubium*. Our research thus provides a framework for the application of CRISPR/Cas9 technology to improve this ornamental crop.

## 1. Introduction

Flower color is an important trait in the commercial value of ornamental plants, which largely determines their market demand [1]. Flower breeders are always looking for novel varieties with original colors. This is also the case for the ornamental bulb plant *Ornithogalum dubium*, which was introduced to the flower industry about 25 years ago from the Western and Eastern Cape of South Africa. Wild *O. dubium* plants generally produce 10- to 30-cm-long flowering stems, each bearing 5 to 25 cream, yellow or orange flowers with dark green or brown centers. This species is grown commercially for bulbs, cut flowers and potted flowers in the United States, Europe and Israel [2,3]. Over the years, as the market for *O. dubium* has grown, the demand for novel colors such as red and other favorable traits such as improved resistance to soft rot has increased worldwide.

The improvement of crop plants with novel desirable traits has relied heavily on the use of classical breeding, random mutagenesis, somatic hybridization and, to a lesser extent, on transgenic methods. The new class of genome-editing technology, CRISPR/Cas9, was developed in model plant systems and is now being applied to dozens of crop species [4]. This novel technology permits generating mutations at a chosen site and complementing desired DNA fragments taking advantage of the double breaks introduced by Cas9 and the plant repair machinery [5]. It enables precise modification of the plant genome via knockout of genes or by enabling genes to gain new functions. Studies in several plant systems have demonstrated that important traits can be manipulated by altering a single gene, or small suite of genes, with CRISPR/Cas9-mediated gene editing. Striking examples are that of plant color that has been obtained by gene editing of anthocyanin biosynthetic genes such as chalcone isomerase (CHI) in rice (Oryza sativa), flavanone-3-hydroxylase (F3H) in carrots (*Daucus carota*) and dihydrofavonol-4-reductase-B (DFR-B) in Japanese morning glories (*Ipomoea nil*). These genes were modified to produce mutants with golden hulls [6], purple-colored calluses [7] and white flowers [8], respectively. Carotenoid biosynthetic genes, like carotenoid isomerase (CRTISO), phytoene synthase 1 (PSY1) and beta-carotene hydroxylase 2 (CrtR-b2) in tomatoes, have also been edited using the CRISPR/Cas9 system to yield a series of novel variations [9,10]. Anthocyanins and carotenoids are the pigments that have the strongest effects on flower color. In most of the *O. dubium* cultivars or hybrids, carotenoids are the main pigments responsible for yellow and orange hues [11]. This leads to the hypothesis that, in this genus, the preferred targets for the editing of flower-specific genes belong to the carotenoid pathway. 

Smulders and Arens [12] concluded that there are three challenges when considering CRISPR/Cas9 in a new plant system. These include (i) the need for an effective transformation system and, most importantly, a regeneration protocol, (ii) the need for sufficient knowledge of a causal gene underlying the trait of interest and (iii) the availability of a whole genome sequence or a complete set of transcriptome sequences. For *O. dubium*, a stable transformation and regeneration system has already been established and used to produce hundreds of transgenic plantlets and plants [2,3]. Moreover, it is a relatively straightforward process to identify target genes in the biosynthetic and regulatory pathways of anthocyanin and carotenoid synthesis, because these genes are well-studied and fairly conserved among different plant species [13,14,15,16]. Nevertheless, the genomic resources for *O. dubium* are scarce, thus limiting the pigment-related target sequences available for editing. To overcome that, we decided to produce a corresponding transcriptome catalogue of *O. dubium* leaves and flowers in order to detect flower-specific genes. 

The relevant plant organs for this analysis, leaves and flowers, were utilized to produce cDNA libraries and then sequenced using the Illumina HiSeq 2500 platform. Over 155 million 100-bp paired-end reads were assembled into an extensive transcriptome of 360,689 contigs with an N50 of 1759 bp. This effort identified about 18,660 transcripts that are differentially expressed between leaves and flowers. Moreover, we found that the carotenoid profiles varied greatly between these plant tissues. We further identified a total of 16 unique transcripts (unigenes) involved in the carotenoid biosynthetic pathway. Finally, a potential gene candidate, LCYB/comp146645_c0, presented a significantly higher expression in flowers and may thus be selected as a target for CRISPR/Cas9 genome editing. The availability of this data establishes a framework for the use of CRISPR/Cas9 gene editing to improve the flower color in *O. dubium* and related species.

## 2. Results and Discussion

### 2.1. Transcriptome Sequencing, De Novo Assembly and Functional Annotation of O. dubium Leaves and Flowers

**Figure 1:**
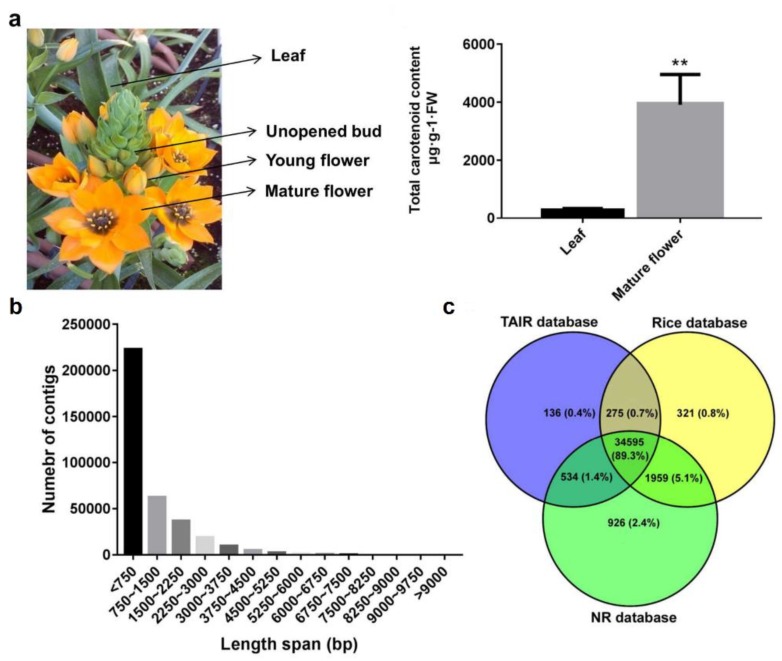
Plant material of *O. dubium* line 5 and its total carotenoid content in leaves and mature flowers (**a**), and the transcriptome sequence information for leaves and flowers of *O. dubium* line 5 (**b**,**c**). (**b**) The distribution of assembled *O. dubium* contigs by length. (**c**) Venn diagram of the distribution and similarity of *O. dubium* contigs, in comparison with sequences from The Arabidopsis Information Resource (TAIR), non-redundant proteins (NR) and rice protein databases.

Two selected *O. dubium* pools (Figure 1a) were used to construct high-throughput parallel RNA-seq libraries. Of these, the flower pool was represented by typical inflorescences at three stages (unopened buds, young flowers and mature flowers), expecting to get collective information on gene transcripts and its expression. Illumina HiSeq 2500 was used for sequencing, yielding 181 million 100-bp raw pair-ends reads with 84.0 and 97.0 million for leaves and flowers, respectively (Table 1). The RNA-sequencing data were deposited in the NCBI Sequence Read Archive (SRA) database under the biological project accession number PRJNA512260 with sample accessions SRR8380847 and SRR8380848. Quality trimming and filtration of two libraries resulted in 72 (85.9%) and 84 (86.3%) million clean reads. Finally, a total of 156 million clean reads were assembled using Trinity, generating 360,689 contigs for the *O. dubium* transcriptome catalogue (Table 1 and File S1).

The average contig length of the *O. dubium* transcriptomes was 956.5 bp with an N50 of 1759 bp (Table 1). The lengths of the assembled sequences ranged from 201 to 16,643 bp; most of the sequences (223,059, 61.8%) were less than 750-bp-long (Figure 1b). All of these transcript data were used to query public genomic databases, including The Arabidopsis Information Resource (TAIR), Rice and non-redundant proteins (NR) databases (Figure 1c), using the BLASTX (Basic Local Alignment Search Tool). The results showed significant hits in three databases, revealing a general similarity of 9.9–10.1% (35,541–38,014) with *O. dubium* contigs (File S2). Most of the contigs (34,595, 89.3%) were found in all three databases. However, some contigs were database-specific; for example, 136 (0.4%) were specific to Arabidopsis, 321 (0.8%) were specific to rice and 926 (2.4%) were specific to NR. Gene ontology (GO) terms via BLAST2GO were assigned to 22,861 contigs based on a sequence similarity with known NR proteins (File S2). Of these GO annotations, the highest representation terms were intracellular (6045), intracellular part (5953), intracellular organelle (5347), intracellular organelle part (1755), cell periphery (1229) and plasma membrane (1035), respectively. The remaining terms were less than 1000, ranging from 1 to 844. In addition, a total of 7582 contigs were mapped to 149 KEGG biological pathways using BLASTX, as demonstrated in File S2. The top 20 represented biological pathways included at least 3000 contigs. The highly represented pathways contained purine metabolism (958), thiamine metabolism (586), a biosynthesis of antibiotics (449), starch and sucrose metabolism (217) and drug metabolism—other enzymes (162). This may be related to the fact that virtually all species in the genus *Ornithogalum* are also medicinal plants, and almost all of their plant tissues have a large number of biosynthetic secondary metabolites with antibiotics activity [2,3,12].

### 2.2. Differential Expression (DE) Transcripts between O. dubium Leaves and Flowers

**Figure 2:**
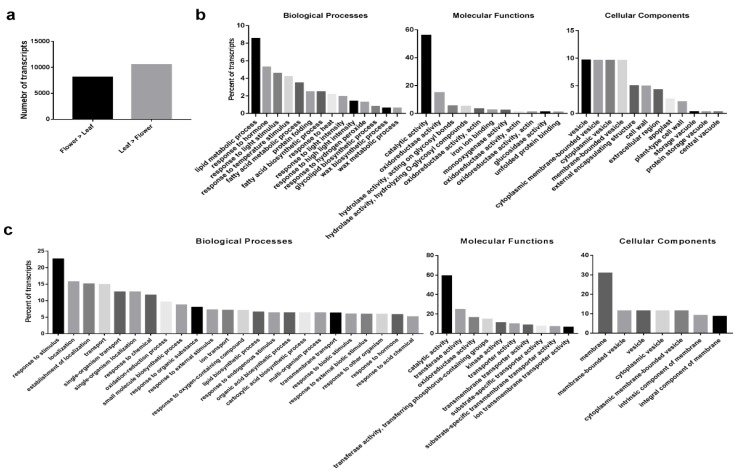
Transcript expression in leaves and flowers of *O. dubium* line 5 (**a**) and gene ontology (GO)-enrichment analysis for upregulated differential expression (DE) transcripts in flowers (**b**) and leaves (**c**). Significantly enriched GO terms (*p* < 0.05) were respectively categorized into three main categories: biological process (BP), cellular component (CC) and molecular function (MF). The *y*-axis shows all the enriched GO terms, while the *x*-axis indicates the percentage of upregulated transcripts in leaves and flowers. Transcripts with percentages less than 0.1% (flowers) and 5.0% (leaves) were not shown.

Gene-expression profiling of *O. dubium* leaves and flowers allowed us to analyze transcripts that are differentially expressed between these tissues. An expressed-transcripts dataset, including 65,031 contigs without the likely artifacts and low-expressed contigs, was initially generated for *O. dubium*. The average contig length was 1499.3 bp with an N50 of 2040 bp (data not shown). A total of 18,660 DE transcripts were found between leaves and combined flowers, showing differential patterns between the vegetative and reproductive tissues (Figure 2a). Of these DE transcripts, 8137 were expressed at higher levels in flowers, as opposed to leaves; 10,523 were expressed at higher levels in leaves. 

GO functional classifications were further analyzed for those upregulated *O. dubium* DE transcripts. Over-representations of GO terms were evaluated to examine which biological processes (BP), molecular functions (MF) and cellular components (CC) were mostly enriched in the leaves or flowers. Overall, the GO-classification distribution of *O. dubium* leaves and combined flowers is very different. As shown in Figure 2b, the most over-expressed flower DE transcripts in the BP category were the lipid metabolic process, response to hormone, response to light stimulus and response to temperature stimulus. The most abundant CC categories that were significantly upregulated in flowers included the vesicle, membrane-bounded vesicle, cytoplasmic vesicle and cytoplasmic membrane-bounded vesicle. Among the notable upregulated MP in the flowers were GO terms related to catalytic activity and oxidoreductase activity. For *O. dubium* leaves, more upregulated GO categories were enriched compared to the flowers. The BP in the set of DE transcripts in the leaves were mostly a response to fungus, response to jasmonic acid, response to red or far-red light and response to bacterium. Other over-represented categories of BP included the alcohol biosynthetic process, fatty acid biosynthetic process, response to abscisic acid and response to wounding. The GO terms for MF that were upregulated in leaves were the catalytic activity, transferase activity and oxidoreductase activity. Most of the DE transcripts represented CC associated with the membrane, membrane-bounded vesicle, vesicle, cytoplasmic vesicle and cytoplasmic membrane-bounded vesicle. 

### 2.3. Common and Specific DE Transcripts between O. dubium Leaves and Flowers

**Figure 3:**
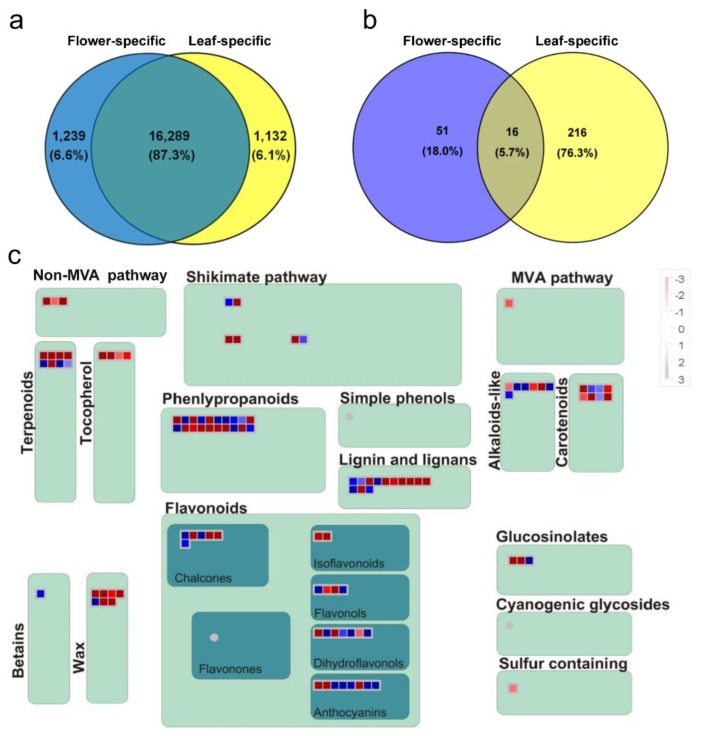
Venn diagram indicating the number of differentially expressed transcripts (**a**) and its corresponding GO-enrichment (**b**) in both flowers and leaves. MapMan regulation overview map (**c**) showing transcript level differences in secondary metabolism of leaves and combined flowers. In color scale, blue represents lower transcript level in flowers, while red represents higher transcript level in comparison with leaves. Note: MVA pathway is an abbreviation for the mevalonate pathway.

A Venn diagram was further constructed to examine the unique and shared DE transcripts between *O. dubium* leaves and flowers. There was a large part of similarity between the two tissues; however, low numbers of specific DE transcripts were also found in each tissue. As shown in Figure 3a, analysis of the DE transcripts revealed that most (16,289, 87.3%) were common to leaves and flowers, while only a few were specific to flowers (1239, 6.6%) or leaves (1132, 6.1%). However, results of the GO-enrichment analysis were contrasted for *O. dubium*-specific and common DE transcripts when the Blast2GO tool was used. Of the 283 enriched GO terms identified (Figure 3b), there was an overlap of 16 (5.7%) terms that were common to both, whereas 51 (18.0%) and 216 (76.3%) terms were specific to the flowers or leaves, respectively. Common GO terms for both the leaves and flowers (false discovery rate (FDR) < 0.05) were mostly annotated as MF and CC, which were mainly related to the catalytic activity and vesicle. For leaf-specific GO terms, a major variety of BP and CC related to the photosynthesis, transport and a response to stimulus, such as the photosynthetic electron transport in photosystem I, photomorphogenesis, response to red or far-red light, photosynthetic electron transport chain, transmembrane transport, response to external stimulus and response to endogenous stimulus, etc., were significantly enriched. For flowers, the most specific BP and CC categories were associated with response to the light/temperature stimulus, response to gibberellin, axis specification and lipid/wax metabolic process. Obviously, a comparative transcriptome profiling of leaves and flowers also allowed to identify large-scale transcripts showing a tissue-specific expression in *O. dubium*. 

Secondary metabolites are often referred to as compounds that are important for the interaction of plants with the environment for adaptation, protection and defense [17]. The MapMan visualization software [18], which is supported by plant-specific gene ontology with minimized redundancy, can offer the possibility to display transcriptome profiling onto diagrams of metabolic pathways and other biological processes. Here, a MapMan regulation overview map analysis was used to focus and visualize gene expression in secondary metabolism pathways between *O. dubium* leaves and flowers. In accordance with the above enriched-flower GO terms, most transcripts in the pathways related with terpenoids (terpene synthase, etc.); tocopherol (phytoene cyclase, etc.); phenylpropanoids (hydroxycinnamoyl-coa shikimate, etc.); lignin and lignans (O-methyltransferase, etc.) and wax (long-chain-alcohol O-fatty-acyltransferase, etc.) appeared to be induced in flowers of *O. dubium* (pooled from different developmental stages) (Figure 3c). Tocopherols and terpenoids are lipid-soluble molecules, which differ only in the degree of saturation of their hydrophobic prenyl side chains. They are implicated in defense against herbivores and pathogens or in the attraction of beneficial organisms, such as pollinators [19]. As essential components of a number of structural polymers, phenylpropanoids also have a similar function to that of tocopherols and terpenoids. The monolignol-derived lignins and lignans can strengthen mechanically the xylem by impregnating the secondary cell wall of both tracheary elements and fibers [20], while cuticle or cuticular waxes can provide mechanical strength and viscoelasticity by covering the outer surfaces of the plants [21]. Both above types of compounds mainly function as a physical barrier against pathogen infection, insect attack, UV exposure and high irradiance, as well as preventing water loss from the plant surface [20,21]. These upregulated metabolites may demonstrate evolutionary adaptations by which plants survive and protect the flowers from various environmental stresses. In addition, the thinner *O. dubium* petal tissues might be enriched in epidermal cells in comparison to the thicker leaf tissues.

Plant secondary metabolites also contribute to specific scents and colors [17]. Except for defense, phenylpropanoids in *O. dubium* flowers may also serve as floral pigments to mediate processes like plant-pollinator interactions. Flavonoids are a large group of phenylpropanoids that provide the plant vivid colors, together with chlorophylls and carotenoids [22]. It includes many compounds like chalcones, isoflavones, flavonols, flavones, dihydroflavonols, anthocyanins and others. Their mixture and blends usually impart the plant with pink to red or blue to purple hues [22]. As shown in Figure 3c, more than half of the flavonoid-related transcript expression of chalcone (chalcone isomerase, etc.); flavonols (oxidoreductase, etc.); dihydroflavonols (dihydrokaempferol 4-reductase, etc.) and anthocyanins (5-aromatic acyltransferase, etc.) were inhibited or reduced in *O. dubium* flowers relative to leaves. This may hint that flavonoids are more important in leaves as defense agents than in flowers. However, most of the transcripts in the carotenoid pathway, including phytoene synthase (PSY), phytoene dehydrogenase (PDS), ζ-carotene desaturase (ZDS), lycopene β-cyclase (LCYB), β-carotene hydroxylase (BCH), etc., were upregulated in flowers. Carotenoids are one of the most widespread pigment groups responsible for bright red, yellow and orange hues in many fruits, vegetables and ornamental plants [13,14]. Previous reports have focused on carotenoid contents in the flower coloration of *O. dubium* and *O. thyrsoides* (two morphologically distinct species). High-pressure liquid chromatography (HPLC) analysis of *O. dubium*, which generally produces bright yellow or dark orange flowers, contained mostly carotenoids and low amounts of chlorophyll, while *O. thyrsoides*, which have white flowers with less intense green/brown centers, do not contain carotenoids but do contain a small amount of chlorophyll located in its base [11]. In line with this, we tried to compare carotenoid contents between flowers and leaves and find the key carotenoids genes that may be responsible for the bright orange flowers of *O. dubium* line 5. 

### 2.4. Carotenoid Contents and Distribution between O. dubium Leaves and Flowers

Carotenoid content and composition of the leaves and fully opened flowers of *O. dubium* were evaluated using high-performance liquid chromatography (HPLC). It is well-known that carotenoids are distributed differentially in photosynthetic (leaves) and nonphotosynthetic tissues (flowers and fruits). As shown in Figure 1a, the total carotenoid content in fresh leaf or flower tissues was significantly different (*p* < 0.01), reaching 294.7 and 3912.8 μg·g-1 fresh weight (FW), respectively. The presence of eight dominant carotenoids, including carotenes (phytofluene, phytoene and β-carotene) and xanthophylls (lutein, β-cryptoxanthin, zeaxanthin, violaxanthin and neoxanthin), were detected. Examination of carotenoid profiles (Table 1) revealed that all eight carotenoids and four unknowns were detected in the mature flower, of which only β-carotene, lutein, zeaxanthin, violaxanthin, neoxanthin and two unknown carotenoids were found in the leaves. A detailed comparison of the individual carotenoid components showed significant differences in their contents (*p* < 0.05) in *O. dubium* leaves in comparison to the flowers. In fresh leaf extracts, lutein and β-carotene are the two main components, whereas, in fresh flowers, zeaxanthin and lutein are predominating. This indicates that carotenoid profiles vary greatly in the photosynthetic (leaf) or nonphotosynthetic (flower) tissues of *O. dubium*.

Lutein and zeaxanthin are derivatives of α- and β-carotene, respectively. Lutein is the most abundant carotenoid in plant photosynthetic tissues, where it plays important role in light-harvesting in photosynthetic membranes, as well as in the protection of the photosystem from photooxidation [23]. Table 2 shows that the concentrations of lutein and β-carotene in leaves were found to be 149.7 and 54.8 μg·g^−1^ FW, accounting for 50.8% and 18.6% of the total carotenoids, respectively. Although the contents of zeaxanthin and lutein in the flowers were much higher than in the leaves, reaching 1561.5 and 880.7 μg·g^−1^ FW, respectively, they only accounted for 40.0% and 22.5% of the total carotenoids. Our results are in accord with the assumption that most plant flowers have distinct carotenoid profiles dependent upon the species and variety [24]. Our findings suggest that lutein and zeaxanthin together represent the main carotenoids pigments in *O. dubium* line 5.

### 2.5. Identification of Key Transcripts in the Carotenoid Biosynthetic Pathway of O. dubium

**Figure 4:**
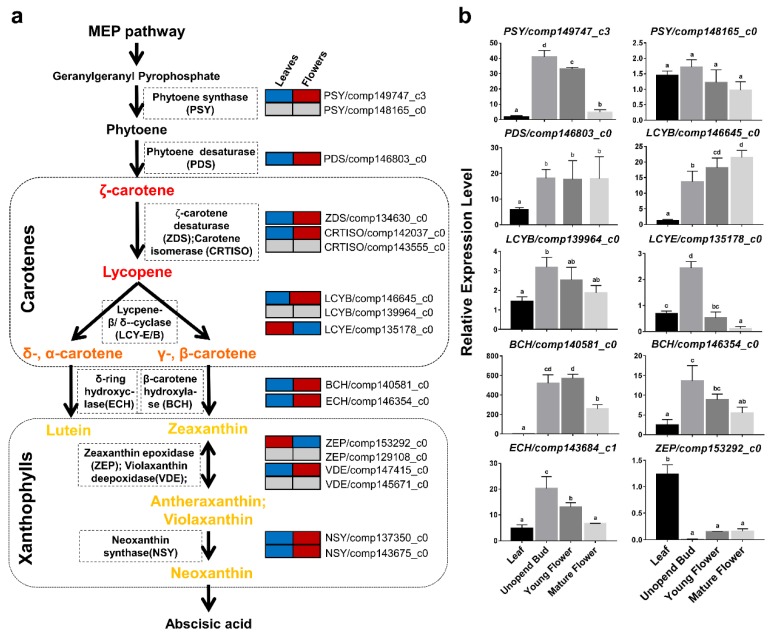
Schematic representation of key transcript expressions in the carotenoid biosynthesis pathway of *O. dubium*. (**a**) Comparison of carotenoid-related transcript expressions between the leaves and flowers based on the transcriptome profiling in *O. dubium* line 5. Gene expression is shown for leaves (left block) and flowers (right block); red represents a higher level of expression of genes, and blue indicates a lower level of expression, while grey means no differences in expression between the two tissues. (**b**) Relative levels of expressions of 10 candidate key transcripts in various tissues (leaves and flowers during three developmental stages) of *O. dubium* line 5. Data from qRT-PCR were normalized with respect to the gene expression of actin3 and presented as the mean with a standard deviation of three biological repeats with three replicates each.

The carotenoid biosynthetic pathway (Figure 4a) has been well-established in the last decades. Carotenoids are produced using precursors mainly generated via the methyl-D-erythritol-4-phosphate (MEP) pathway [23,24]. Colorless carotenoid 15-cis-phytoene is the first one, formed with the head-to-head condensation of two molecules of geranylgeranyl diphosphate catalyzed by a phytoene synthase (*PSY*). Following a series of desaturations by phytoene desaturase (*PDS*) and ζ-carotene desaturase (*ZDS*) and isomerizations by carotenoid isomerase (*CRTISO*) and 15-cis ζ-carotene isomerase (*Z-ISO*) the colorless phytoene is converted to red-colored all-t*rans*-lycopene, which is cyclized by either lycopene ε-cyclase (*LCYE*) or lycopene β-cyclase (*LCYB*) to yield β-carotene (β and β-carotene) or α-carotene (β and ε-carotene), the precursors for β-branch and the α-branch xanthophylls, respectively. Then, hydroxylation of the β-rings or ε-rings by carotene β-hydroxylase (*BCH*) and carotene ε-hydroxylase (*ΕCH*) gives rise to the xanthophylls zeaxanthin and lutein, respectively. Zeaxanthin is epoxidized by zeaxanthin epoxidase (*ZEP*) to yield yellow violaxanthin via the intermediate antheraxanthin. Violaxanthin can also be converted back into zeaxanthin by violaxanthin de-epoxidase (*VDE*). The final step is to convert yellow-colored violaxanthin into neoxanthin, the precursor of abscisic acid (ABA), by neoxanthin synthase (*NSY*). Based on the BLASTX results against the Nr database, we identified 27 carotenoid-related transcripts (Table S1) from the assembled transcriptomes of leaves and combined flowers in *O. dubium*. A total of 16 unique transcripts (unigenes) involved in the carotenoid biosynthesis pathway were further inferred (Figure 4a) after non-redundant analysis. Among them, only single unigenes were aligned to *PDS*, *ZDS*, *LCYE*, *ECH* and *ZEP*, while two unigenes were found for *PSY*, *LCYB, BCH* and *VDE*. 

While the chloroplasts in green tissues maintain a somewhat constant carotenoid composition to ensure proper functionality of the photosynthetic apparatus, a carotenoid profile of chromoplasts, in non-photosynthetic tissues such as flowers and fruits, is diverse and usually very different from the profile found in green tissues. Distinct regulatory mechanisms in the different tissues control carotenoid biosynthesis, storage and degradation to ensure the accumulation of the carotenoid profile typical for each cell type. One of the mechanisms that maintain a separate regulation for each tissue is the existence of a sets of genes with each member acting in different types of organelles. For instance, in tomatoes, three sets of carotenogenic biosynthesis genes: *PSY*, *LCYB* and *BCH* participate in carotenoid biosynthesis. One member of each set is predominantly active in chloroplasts, while another member is mainly active in chromoplasts. The enzymes *PSY2* and *CRTR-b1* (a carotene β-hydroxylase) are expressed preferentially in photosynthetic tissues (leaves), while *PSY1* and *CRTR-b2* are expressed more strongly in flowers and fruits [25,26,27,28]. In the case of *LCYB*, *CRTL-b1* (*LCYB1*) is expressed in leaves and at very low levels in flowers, whereas *CRTL-b2* (*CYCB*) is expressed strongly in flowers and at very low levels in fruits [29,30]. Here, we focused on the transcription of chloroplast- and chromoplast-typical carotenoid genes in foliar and floral tissues of *O. dubium*. Mining the data from the transcriptome, the expression of 16 unique unigenes was found in leaves and combined flowers (Figure 4a and Table S1). Thirteen out of 16 unigenes, covering the key genes in the carotenoid biosynthesis pathway, were differentially expressed (DE) between leaf and flower pools. Except for *LCYE/comp135178_c0* and *ZEP/comp153292_c0*, all other 11 DE unigenes were expressed at higher levels in combined *O. dubium* flowers (red), as opposed to leaves (blue) (Figure 4a). Pairs of genes encoding *PSY*, *LCYB* and *BCH* were detected in the *O. dubium* transcriptome. Similar to tomatoes, chromoplast-specific *PSY1*, *CRTR-b2* (*LCYB2*) and *CRTL-b1*, the *PSY/comp149747_c3*, *LCYB/comp146645_c0*, *BCH/comp140581_c0* and *BCH/comp146354_c0*, presented higher expression levels in *O. dubium* flowers. No chloroplast-specific highly expressed genes encoding *PSY*, *LCYB* or *BCH* were observed in *O. dubium* leaves. *PSY/comp148165_c0* was expressed similarly in leaves and flowers, with low expression levels in both (Table S2). The expression of *LCYB/comp139964_c0* was higher in flowers but not significantly different from that of the leaves. We suggest that the high expression levels of *PSY/comp149747_c3*, *LCYB/comp146645_c0*, *BCH/comp140581_c0* and *BCH/comp146354_c0* contribute to the higher levels of carotenoids in *O. dubium* mature flowers in comparison to the leaves. Possibly, the high expression of the upstream genes *PDS/comp146803_c0*, *ZDS/comp134630_c0* and *ECH/comp143684_c1* may also contribute to the high levels of carotenoids in the flowers. For instance, in tomatoes, *PDS* was shown to be preferentially expressed in chromoplast-containing tissues, as revealed by the functional analysis of its promoter [31]. While the biosynthesis of zeaxanthin in the flowers might be explained by the high expression of structural genes upstream in the biosynthesis pathway, its accumulation could be explained by the lower expression of the downstream gene *ZEP/comp153292* in flowers, in comparison to leaves. The higher expression of *LCYE/comp135178_c0* in leaves in comparison to flowers could be associated with a higher flux of the biosynthetic pathway toward α-branch products, such as lutein, in the leaves in comparison to the flowers, where β-branch carotenoids are more abundant. 

To validate some of the RNA-seq results, a qRT-PCR analysis was conducted on *O. dubium* plant samples (leaves and flowers at different stages). Genes in the qRT-PCR analysis (Figure 4b) included ten carotenogenic biosynthesis transcripts (*PSY*, *LCYB*, *PDS*, *LCYE*, *BCH*, *ECH* and *ZEP*), all of which showed results in agreement with the RNA-seq. Diverse gene expression patterns were observed at the unopened buds, young flowers and mature flowers of *O. dubium*. Transcription levels of carotenoid biosynthesis genes have been shown to be tightly associated with the carotenoid content and composition in many plant tissues; however, there are additional factors that regulate carotenoid accumulation [32]. Interestingly, *PSY/comp149747_c3*, *LCYE/comp135178_c0*, *BCH/comp140581_c0*, *BCH/comp146354_c0* and *ECH/comp143684_c1* transcription levels decreased during flower development. The transcription of *PDS/comp146803_c0* and *ZEP/comp153292* remained constant, and only the transcription of *LCYB/comp146645_c0* increased during flower development.

Lycopene is a carotenoid that provides red color to plant organs such as tomato fruits. It is an intermediate product which accumulates as a result of the activation of the first steps of the carotenoid biosynthesis pathway up to lycopene and the inhibition of the enzymatic steps that follow, namely the cyclization of lycopene, as was shown for tomato fruits [30,33]. In other plants, mutations associated with the decreased activity of *LCYB* were shown to cause lycopene accumulation, such as in red watermelons [34,35], papaya [36], red grapefruits [37] and red oranges [38]. In addition, tomato plants carrying a null mutation in the tomato chromoplasts-specific *LCYB*, or transgenic tomato plants in which this gene is silenced, showed an accumulation of lycopene in their flowers, a tissue that does not normally accumulate lycopene [30]. Therefore, we hypothesize that the lycopene cyclase genes identified at present in *O. dubium* may serve as CRIPSPR-editing targets to generate red flower varieties.

## 3. Materials and Methods

### 3.1. Plant Materials

*O. dubium* line 5 (Figure 1) is an orange clone of a potted plant that has been developed in a a recent breeding program that aimed to produce new *O. dubium* phenotypes. Line 5 is the result of a successful cross between two orange *O. dubium* accessions at the Agricultural Research Organization (ARO), The Volcani Center, Bet Dagan, Israel. It was chosen for its bright orange flowers, rich inflorescence and compact size, which were suitable for a commercial potted plant. Since then, line 5 has been cloned in our lab using common micropropagation protocols, and plantlets were transferred to the greenhouse and grown under ambient conditions (5–25 °C) for the autumn–spring season. After two years, bullets (circumferences of about 3 cm) were planted in a medium of coconut peat, volcanic tuff particles and compost (V/V/V = 50:40:10). The plants were fertilized regularly with Shefer liquid fertilizer (N:P:K = 59:35:94 g/L; Dshanim, Israel). 

### 3.2. RNA Extraction, Pair-End Library Construction and Sequencing

Tissue samples (Figure 1a), including the fully opened younger green leaves of each leaf rosette, the unopened buds, the young flowers and the mature flowers from each inflorescence, were separately collected from three different plants. Each plant inflorescence contained 12–20 flowers in different developmental stages; these were used as replicates. All samples were divided into three sections for RNA sequencing, carotenoid extraction and quantitative PCR experiments before frozen in liquid nitrogen and stored in a −80 °C refrigerator. For RNA extraction, pair-end library construction and sequencing, leaves and flowers from three plants and three replicates each were respectively combined as two pools to emphasize the gene differences between the leaves and flowers in *O. dubium*. Total RNA of each sample was extracted using the RNeasy Mini Kit (Qiagen, Hilden, Germany), according to the manufacturer’s instructions. RNA purity and integrity were verified using the Agilent 2100 Bioanalyzer (Agilent Scientific, Santa Clara, CA, USA) with a minimum RNA integrity number value of 7.0. RNA libraries were prepared and sequenced at the Genome Center, Life Sciences and Engineering Infrastructure Center, Technion, Haifa, Israel. Two independent pair-end RNA-seq libraries, with a length of 100 nucleotides, were generated for the leaves and flowers using the Illumina HiSeq2500 and TrueSeq protocols (Illumina, San Diego, CA, USA).

### 3.3. Sequence Processing, Transcriptome Assembly and Functional Annotation

Raw reads were subjected to a cleaning procedure using the FASTX toolkit, including (i) trimming read-end nucleotides with quality scores < 30 using a fastq_quality_trimmer and (ii) removing read pairs if either one had less than 70% base pairs with a quality score ≤ 30 using a fastq_quality_filter. After processing and cleaning, we had a total of 156 million cleaned reads; the reads were assembled de novo using Trinity software [39], version trinityrnaseq_r20131110, with default parameters and a 25mer k-mer size. The likely contig artifacts and low-expressed contigs were filtered out as follows: (i) abundance estimates were calculated for each contig using the RSEM software package [40], (ii) only contigs representing more than 0.5 fragments per feature kilobase per million reads mapped (FPKM) and 1.0% of the per-component (IsoPct) expression levels were retained. 

BLASTX was used to annotate the assembled contigs against the databases of Arabidopsis, *Oryza sativa* and NR with an E-value cut-off of 10E-5. Blast2GO [41] was used to obtain GO annotations, and Ontologizer [42] was used to perform GO functional classification. The contigs were also compared with the KEGG database using BLASTX with an E-value cut-off of < 10E-3, to assign the detected assembled sequences to biological pathways.

### 3.4. Differential Expression (DE) of Transcripts and GO-Enrichment Analysis

The clean reads from each library were aligned separately with the transcriptome catalogue using a Bowtie aligner [43]. The abundance estimation was calculated via expectation maximization, using the run_RSEM_align_n_estimate.pl Perl script, following the Trinity (version trinityrnaseq_r20131110) protocol [44]. Differential-expression analysis was performed with the edgeR package with dispersion parameters between 0.1 and 0.4 [45] based on the count estimations for each contig. Transcripts that were more than twofold differentially expressed with an adjusted P-value of no more than 0.05 [46] were considered differentially expressed. DE transcripts in leaves or flowers were analyzed for GO-category enrichment relative to all transcriptome databases using the Fisher’s exact test of Blast2GO software [41]. A false discovery rate (FDR) with a corrected P-value of less than 0.05 was set as the threshold. DE transcripts of leaves and flowers were displayed on the diagram of a regulation overview map using MapMan [47] and TAIR10 mapping (Ath_AGI_TAIR9_Jan2010).

### 3.5. Carotenoid Extraction and HPLC Analysis

The second section of tissues samples that were collected was used for carotenoid extractions. Carotenoids were extracted from three biological repeats (plants) of each tissue, each containing at least 3 flowers and 3 leaves. Carotenoid extractions followed previously described protocols [48] with some changes: frozen leaf tissue (~80 mg) or petal tissue (~15 mg) was ground in 1-ml acetone, and extract was collected and filtered. This was repeated, and acetone extracts were combined until tissue was colorless. The pigments’ extract was dried under a stream of nitrogen. For extracts of leaf tissues, pigments were dissolved in methanol:acetone (2:1) solution and used in HPLC analysis. Dry extracts of petals were subjected to saponification by addition of ethanolic KOH (6%) and gentle agitation for 16 hours in the dark. Carotenoids were extracted with an equal volume of diethyl-ether and a 0.2 volume of saturated NaCl solution. Ether phase was collected, dried and dissolved in methanol:acetone (2:1) for HPLC analysis. All procedures were carried out under dim light, and samples were kept in -20℃ and under anaerobic atmosphere. 

HPLC was carried out with a Waters HPLC system (Water Corporation, Milford, MA, USA) equipped with an HPLC 600 pump and a 996 Photodiode Array (PDA) detector and a 717 plus autosampler. A C30 column (YMCA, 5 µm, 4.6 × 250 mm) coupled to a C30 guard column system (YMCA) was used. Separation protocol is based on a previously described method [49]. A constant flow of 1 mL/min was applied with an initial solvent composition of 90% methanol, 5% water and 5% of methyl *tert*-butyl ether (MTBE), changing linearly to 95% methanol and 5% MTBE during 12 min. During the next 8 min, the composition was changed linearly to 86% methanol and 14% MTBE and followed by 75% methanol and 25% MTBE for 10 min. Final step of 50% methanol and 50% MTBE was reached at 50 min and maintained for an additional 20 min. Initial composition was established during 2 min, and column was equilibrated for 20 min before the next injection. The data was recorded at 250–600 nm and analyzed by the “Empower” software (Water Corporation, Milford, MA, USA). Carotenoids were identified by their absorption spectra and retention times. β-carotene standard was obtained from Sigma-Aldrich, phytoene standard was obtained from CaroteNature (Switzerland) and β-cryptoxanthin and lutein from Extrasynthase (France). Standard for zeaxanthin was extracted from *Escherichia coli* engineered to accumulate zeaxanthin [50]. All carotenoid peaks were normalized to correct for their specific mass extinction coefficients [51] in relation to β-carotene (= 1), using xanthophylls (1), β-cryptoxanthin (1.086), phytofluene (1.920) and phytoene (2.074). Total carotenoid content, presented as μg g^−1^ fresh weight (FW), was determined based on β-carotene calibration curves prepared with authentic standards.

### 3.6. Quantitative PCR Analysis

Total RNA was extracted for each sample as described in pair-end RNA-seq library preparation and sequencing. Key genes in the qRT-PCR analysis included PSY, PDS, LCYB, LCYE, BCH, ECH and ZEP. The gene-specific PCR primer sets designed by Primer 3.0 are listed in Supplementary Table S1. The gene *actin3* was used as an internal reference gene. SYBR green-based qRT-PCRs were performed for all genes with biological triplicates using the ABI StepOne Plus system (Applied Biosystems, Foster City, CA, USA). Relative transcript levels for each sample were calculated using the delta-delta CT (ΔΔCT) method [52]. 

## 4. Conclusions

In *O. dubium*, three lycopene cyclase genes, including two *LCYB* (comp146645_c0 and comp139964_c0) and one *LCYE* (comp135178_c0), were successfully identified, and their expression was indicated by RNAseq and validated by qRT-PCR analysis. Our qRT-PCR results confirmed that *LCYB/comp146645_c0*, like tomato *CRTR-b2* (*CYCB*), has a higher expression in flowers than in leaves. The other *lycopene β-cyclase*, *LCYB/comp139964_c0*, unlike tomato *CRTR-b1*, is constitutively expressed in both leaves and flowers. We suggest that *LCYB/comp146645_c0* could be selected as a CRISPR-Cas9 target to implement lycopene accumulation in *O. dubium* flowers. Since *LCYB/comp139964_c0* and *LCYE/comp135178_c0* are active in leaves, and apparently required for carotenoid biosynthesis for photosynthesis, we do not think that disturbing their function by the CRISPR-Cas9 strategy is a viable option. 

## Figures and Tables

**Table 1:** Raw and clean reads for leaves and combined flowers of *O. dubium* line 5.

Sample	Leaves	Flowers
Raw reads	83,956,119	97,041,800
Clean reads (%)	72,125,398 (85.9%)	83,738,661(86.3%)
Total clean reads (%)	155,864,059 (86.1%)	
Total assembled bases	344,991,254 bp	
Sequence number	360,689	
Median contig length	494 bp	
Length range	201–16,643 bp	
Mean length	956.5 bp	
N50	1759 bp	
GC content	41.1%	

**Table 2:** Carotenoids contents (μg·g^−1^ fresh weight (FW)) in the leaves and mature flowers of *O. dubium* line 5.

Carotenoid Composition	Leaves (Mean ± Std)	Flowers (Mean ± Std)
Phytoene	0.0	20.7 ± 5.6
Phytofluene	0.0	16.0 ± 9.0
β-carotene	54.8 ± 22.0	80.2 ± 22.9 ** ^b^
Lutein	149.7 ± 35.4	880.7 ± 192.8 **
β-cryptoxanthin	0.0	117.6 ± 39.1
Zeaxanthin	9.0 ± 3.3	1,561.5 ± 304.5 ***
Violaxanthin	12.5 ± 5.8	128.8 ± 59.0 *
Neoxanthin	2.7 ± 0.7	147.2 ± 94.1 *
^a^ UK2	24.2 ± 9.1	9.4 ± 8.9
UK3	15.0 ± 5.8	21.3 ± 4.9
UK5	0.0	528.6 ± 226.9
UK11	0.0	159.0 ± 44.0
others	27.0 ± 11.5	241.8 ± 53.9 **

^a^ UK means unknown carotenoids; ^b^ the asterisk indicates a significant difference (at *p* < 0.05 *, 0.01 ** or 0.001 ***) between the leaves and flowers.

## Supplementary Materials

The following are available online at https://www.mdpi.com/2223-7747/9/4/540/s1: File S1: Assembled contigs of O. *dubium* leaves and flower, File S2: Function annotations of O. *dubium* assembled contigs, Table S1: The key transcripts involved in the carotenoid biosynthesis pathway and its expressions in the leaves and combined flowers of *O. dubium*. Table S2: List of qPCR primer pairs used to validate the gene expressions in the leaves and flowers of *O. dubium*.
