# Supplementary material for: Transcriptome Profiling of Ornithogalum dubium Leaves and Flowers to Identify Key Carotenoid Genes for CRISPR Gene Editing
Source: Plants (Basel). 2020 Apr 21;9(4):540. doi: 10.3390/plants9040540 (PMC7238968; doi:10.3390/plants9040540)
Supplement: Supplementary file 1 [file plants-09-00540-s001.zip › Supplementary plants-767988/plants-767988-TableS1-S2.docx]

**Table S1.** The key transcripts involved in the carotenoid biosynthesis pathway and its expressions in leaf and combined flowers of *O. dubium*.

| Genes Name | Transcripts ID | Flower transcripts count | Leaf  Transcripts count | Flower. TMM.FPKM | Leafs2. TMM. FPKM | LogFC (Leaf/Flower) | P-Value | FDR |
| --- | --- | --- | --- | --- | --- | --- | --- | --- |
| *PSY* | comp148165_c0_seq1 | 194.65 | 228.84 | 3.034 | 3.502 | 0.2101 | 0.7593 | 0.8816 |
|  | comp148165_c0_seq2 | 152.73 | 256.05 | 2.267 | 3.724 | 0.7205 | 0.2884 | 0.4896 |
|  | comp149747_c3_seq1 | 18159.16 | 3370.57 | 133.928 | 24.179 | -2.4511 | 0.0004 | 0.0022 |
|  | comp149747_c3_seq2 | 24571.99 | 4286.13 | 193.139 | 32.78 | -2.5410 | 0.0002 | 0.0015 |
|  | comp149747_c3_seq3 | 31724.26 | 4923.94 | 248.097 | 37.471 | -2.7093 | 0.0001 | 0.0007 |
| *PDS* | comp146803_c0_seq1 | 19097.54 | 4524.76 | 187.827 | 43.394 | -2.0991 | 0.0020 | 0.0095 |
|  | comp146803_c0_seq2 | 16702.03 | 3822.62 | 165.034 | 36.832 | -2.1489 | 0.0016 | 0.0078 |
| *ZDS* | comp134630_c0_seq1 | 58868.76 | 6353.84 | 597.101 | 62.865 | -3.2334 | 0.0000 | 0.0001 |
|  | comp140237_c0_seq1 | 3791.25 | 853.23 | 48.891 | 10.764 | -2.1735 | 0.0015 | 0.0073 |
|  | comp140237_c0_seq2 | 4091.54 | 841.33 | 52.184 | 10.498 | -2.3041 | 0.0008 | 0.0042 |
| *ZISO* | comp140237_c0_seq1 | 3791.25 | 853.23 | 48.891 | 10.764 | -2.1735 | 0.0016 | 0.0073 |
|  | comp140237_c0_seq2 | 4091.54 | 841.33 | 52.184 | 10.498 | -2.3041 | 0.0007 | 0.0042 |
| *LCYB* | comp139964_c0_seq1 | 13331.82 | 8061.26 | 161.064 | 95.193 | -0.7475 | 0.2554 | 0.4512 |
|  | comp146645_c0_seq1 | 1287.02 | 92.0 | 20.65 | 1.445 | -3.8261 | 0.0000 | 0.0000 |
| *LCYE* | comp135178_c0_seq1 | 1689.04 | 5381.72 | 21.271 | 66.296 | 1.6502 | 0.0140 | 0.0487 |
| *BCH* | comp140581_c0_seq1 | 119561.91 | 2739.13 | 1573.15 | 35.272 | -5.4696 | 0.0000 | 0.0000 |
|  | comp146354_c0_seq1 | 30118.66 | 9419.92 | 317.785 | 96.993 | -1.6985 | 0.0114 | 0.0411 |
| *ECH* | comp143684_c1_seq2 | 847.75 | 597.58 | 8.346 | 5.737 | -0.5255 | 0.4290 | 0.6320 |
|  | comp143684_c1_seq7 | 1976.42 | 1088.21 | 19.534 | 10.489 | -0.8825 | 0.1819 | 0.3581 |
|  | comp143684_c1_seq8 | 1335.46 | 70.84 | 13.252 | 0.683 | -4.2521 | 0.0000 | 0.0000 |
| *ZEP* | comp153292_c0_seq2 | 1325.32 | 26354.02 | 12.868 | 249.446 | 4.2921 | 0.0000 | 0.0000 |
| *VDE* | comp145671_c0_seq1 | 862.27 | 2303.97 | 10.038 | 26.192 | 1.3966 | 0.0367 | 0.1067 |
|  | comp145671_c0_seq2 | 33.1 | 48.35 | 0.654 | 0.94 | 0.5172 | 0.4917 | 0.6870 |
|  | comp145671_c0_seq3 | 761.02 | 1592.76 | 6.496 | 13.238 | 1.0440 | 0.1161 | 0.2584 |
|  | comp147415_c0_seq1 | 216 | 613 | 2.492 | 6.898 | 1.4826 | 0.0291 | 0.0887 |
| *NSY(ABA4)* | comp137350_c0_seq1 | 13991.07 | 333.44 | 264.067 | 6.198 | -5.4140 | 0.0000 | 0.0000 |
|  | comp143675_c0_seq2 | 10099.57 | 10.65 | 131.481 | 0.133 | -9.8479 | 0.0000 | 0.0000 |

**Table S2.** List of qPCR primer pairs used to validate gene expression in leaves and flowers of *O. dubium*.

| Genes Name | Sequence ID | Forward Primer | Reverse Primer | Length (bp) | Tm (℃) |
| --- | --- | --- | --- | --- | --- |
| *PSY* | comp149747_c3_seq1 | GTGACCGACAAGTGGAGGAACT | CCACCTACTGGCTTGGCTGA | 108 | 63.0 |
| *PSY* | comp148165_c0_seq1 | TGGGGTCTTCAGAGGCTGTT | AGGGAGGTTGCCACTCACTT | 137 | 60.0 |
| *PDS* | comp146803_c0_seq1 | TATTATTGGAGGCGAGGGATGT | TTCTGCACGTTGGGATAAGCT | 118 | 60.0 |
| *LCYB* | comp146645_c0_seq1 | TGAGAATGGGGTTCGGTTTC | AGGCTCGCCGTTATGTTTG | 105 | 59.0 |
| *LCYB* | comp139964_c0_seq1 | AACCGCAAGAATCTCAAGTCC | AGCCTCAATAGTGACCCCATC | 141 | 60.0 |
| *LCYE* | comp135178_c0_seq1 | TTGGATGTGGCAGGGATTTC | ATGCTGTGCGGTGCGATTA | 93 | 58.0 |
| *BCH* | comp140581_c0_seq1 | CAGATGCGGATCTCGGAAGC | TGCCAGGCGAATCGGTAGTA | 146 | 61.0 |
| *BCH* | comp146354_c0_seq1 | TTATTCCAAGGGGATTTTAGCG | TCATCGCAGCCACATACTTTT | 137 | 58.0 |
| *ECH* | comp143684_c1_seq8 | GCAGTGGTTCCGTCCCTTCATAA | GTTCACCGCAGCCCCGTTC | 123 | 59.6 |
| *ZEP* | comp153292_c0_seq2 | AGTGAACATGGAAGCTGGATTGT | GGCATAGGACGGGTGGAAA | 90 | 60.0 |
| *Actin 3* | comp145152_c0_seq10 | CCTCAACCCCAAGGCAAAC | CGACCACTGGCATACAAAGAAA | 114 | 59.5 |
